# Cerebral Toxoplasmosis Mimicking Subacute Meningitis in HIV-Infected Patients; a Cohort Study from Indonesia

**DOI:** 10.1371/journal.pntd.0001994

**Published:** 2013-01-10

**Authors:** A. Rizal Ganiem, Sofiati Dian, Agnes Indriati, Lidya Chaidir, Rudi Wisaksana, Patrick Sturm, Willem Melchers, Andre van der Ven, Ida Parwati, Reinout van Crevel

**Affiliations:** 1 Department of Neurology, Hasan Sadikin Hospital, Bandung, Indonesia; 2 Department of Clinical Pathology, Hasan Sadikin Hospital, Bandung, Indonesia; 3 Health Research Unit, Faculty of Medicine, Universitas Padjadjaran, Bandung, Indonesia; 4 Department of Internal Medicine, Hasan Sadikin Hospital, Bandung, Indonesia; 5 Department of Medical Microbiology, Radboud University Medical Centre, Nijmegen, The Netherlands; 6 Department of Medicine, Radboud University Medical Centre, Nijmegen, The Netherlands; University of Washington, United States of America

## Abstract

**Background:**

HIV-associated subacute meningitis is mostly caused by tuberculosis or cryptococcosis, but often no etiology can be established. In the absence of CT or MRI of the brain, toxoplasmosis is generally not considered as part of the differential diagnosis.

**Methodology/Principal Findings:**

We performed cerebrospinal fluid real time PCR and serological testing for *Toxoplasma gondii* in archived samples from a well-characterized cohort of 64 HIV-infected patients presenting with subacute meningitis in a referral hospital in Indonesia. Neuroradiology was only available for 6 patients.

At time of presentation, patients mostly had newly diagnosed and advanced HIV infection (median CD4 count 22 cells/mL), with only 17.2% taking ART, and 9.4% PJP-prophylaxis. CSF PCR for *T. Gondii* was positive in 21 patients (32.8%). Circulating toxoplasma IgG was present in 77.2% of patients tested, including all in whom the PCR of CSF was positive for *T. Gondii*. Clinically, in the absence of neuroradiology, toxoplasmosis was difficult to distinguish from tuberculosis or cryptococcal meningitis, although CSF abnormalities were less pronounced. Mortality among patients with a positive CSF *T. Gondii* PCR was 81%, 2.16-fold higher (95% CI 1.04–4.47) compared to those with a negative PCR.

**Conclusions/Significance:**

Toxoplasmosis should be considered in HIV-infected patients with clinically suspected subacute meningitis in settings where neuroradiology is not available.

## Author Summary

If HIV-infected patients present with seizures, focal neurological symptoms or confusion, a CT-scan or MRI of the brain is normally made. If mass lesions are found (and the CD4 cell count is sufficiently low), cerebral toxoplasmosis is suspected, and often treated empirically. However, some of the symptoms of cerebral toxoplasmosis may mimic those of subacute meningitis. Therefore, in settings where no cerebral imaging can be performed, HIV-associated cerebral toxoplasmosis may be under-diagnosed. We retrospectively looked for toxoplasmosis in a cohort of HIV-infected patients presenting with subacute meningitis in an Indonesian hospital, where neuroradiology was not available for most patients. Patients mostly came with newly diagnosed and advanced HIV infection and few were on HIV-treatment or PJP-prophylaxis. Molecular testing of cerebrospinal fluid (CSF) was positive for *Toxoplasma gondii* in 32% of patients, serology was positive in 78%. Clinically, in the absence of neuroradiology, toxoplasmosis was difficult to distinguish from tuberculosis or cryptococcal meningitis. A positive CSF *T. gondii* PCR was associated with a two-fold increased mortality. We conclude that toxoplasmosis should be considered in HIV-infected patients with clinically suspected subacute meningitis in settings where neuroradiology is not available.

## Introduction

In settings of Africa and Asia, the most common cause of subacute meningitis in patients with advanced HIV infection is either tuberculous or cryptococcal infection [1], [2]. However, in many patients, the etiology of subacute meningitis cannot be established [1], [3]. In line with a large retrospective cohort of adult meningitis patients in South Africa, where 52.8% had no definite diagnosis despite extensive microbiological testing [1], we could not identify the causative pathogen in 48.9% of HIV-infected meningitis patients in an Indonesian setting [4].

Toxoplasmosis is a common and serious central nervous system (CNS) infection in patients with advanced HIV infection [5]–[8], although its incidence has decreased with introduction of antiretroviral treatment (ART) [6], [9]. Cerebral toxoplasmosis mostly presents as cerebral mass lesions with headache, confusion, fever, lethargy, seizures, cranial nerve palsies, psychomotor changes, hemiparesis and/or ataxia [10]. Some of these symptoms may also mimic meningitis, but cerebral toxoplasmosis is generally not considered as a differential diagnosis of subacute meningitis in HIV-infected patients. This is especially the case in low-resource settings where no CT or MRI can be performed. We have therefore examined if toxoplasmosis can be diagnosed in HIV-infected patients presenting with subacute meningitis of unknown origin in Indonesia, using cerebrospinal fluid (CSF) PCR for *T. gondii*.

## Methods

### Ethics statement

Anonymized CSF and blood samples were used from an already-existing hospital collection, from a cohort of patients collected as part of a project ‘Optimization of diagnosis of meningitis’, approved by the Ethical Committee of Hasan Sadikin Hospital/Medical Faculty of Universitas Padjadjaran, Bandung, Indonesia (No. 85/FKUP-RSHS/KEPK/Kep/EC/2006). As this study was done using already existing sample collection, no separate consent was asked for this study. HIV testing is done routinely with oral informed consent for all patients with suspected meningitis in Hasan Sadikin hospital, after 24% were found HIV-positive in a previous cohort study of 185 patients in the same hospital [4]. Consent is obtained from closest relatives (husband/wife or parents) for those patients who are unstable or unconscious at time of presentation. With approval from the ethical committee HIV testing was done anonymously afterwards for those who had died before consent could be obtained.

### Setting and patients

We included adult patients presenting with suspected meningitis at Hasan Sadikin Hospital, the top referral hospital for West Java Province, Indonesia, between December 2006 and October 2010. Clinical data including outcome was recorded in individual case report form. Definite TB meningitis was diagnosed if CSF culture or real time PCR were positive for *M. tuberculosis*, cryptococcal meningitis if either CSF India Ink examination, culture or cryptococcal antigen testing were positive, and toxoplasmosis if CSF *T. gondii* PCR was positive. HIV testing is done routinely for patients presenting at this hospital, but cerebral CT-scanning is rarely done in this setting and is not covered by the government health insurance for the poor.

### Laboratory examinations

CSF cell count and differentiation, protein and glucose were measured. CSF microscopy was done for cryptococci, acid-fast bacilli and bacterial pathogens. CSF was cultured for *Mycobacterium tuberculosis* (solid Ogawa and liquid MB-BacT, Biomerieux), bacterial pathogens (blood agar, chocolate agar, and brain-heart infusion) and fungi (Sabouraud). Cryptococcal antigen (CALAS, Meridian Diagnostics) testing was done on CSF samples following the manufacturer's instructions. Five to 7 ml CSF samples were used for molecular testing. After centrifugation of CSF samples at 3000×g for 10 minutes, DNA was extracted from 200 µl of CSF sediment by using QIAmp DNA mini kit (Qiagen, USA). CSF *M. tuberculosis* real time PCR was done using *IS6110*, a repeated insertion sequence specific for *M. tuberculosis*, as a target [11]. Measurement of CD4-cell count for HIV-patients only became available during the time of the study and was measured only for those who survived for more than 4 days. Real time PCR for *T. gondii*, using the multicopy B1 gene of the *T. gondii* as the target as described elsewhere [12], was performed to archived CSF samples at Radboud University Nijmegen Medical Centre. CSF specimens from 22 HIV-negative meningitis patients (16 with definite TB meningitis, 2 with bacterial meningitis, and 4 with no definite diagnosis), and nine patients with non-infectious CNS diseases, all recruited at Hasan Sadikin Hospital, were used as controls for *T. gondii* PCR. These samples were collected during the study period over a similar time scale compared to the case CSF samples. Toxoplasma immunoglobuline G (toxoplasma IgG) were measured by electro chemiluminescent assay (ECLIA, Elecsys, Roche) in archived serum samples of patients included in the study.

### Data analysis and statistics

Characteristics of patients with definite tuberculosis, cryptococcosis and toxoplasmosis were compared using Chi-square test for proportions and Mann-Whitney U test for continuous variables. Progression to death using 2-month mortality data was examined by Kaplan–Meier estimates.

## Results

During the period, 401 patients presented with clinical meningitis, 76 were diagnosed with HIV infection, and 64 had archived CSF samples and were included in this study. Patients included in the study presented after a median 7 days, with meningismus (86.0%), headache (80.8%), lowered consciousness (33.3%), fever (28.8%), hemi- or tetraparesis (28.6%), cranial nerve palsies (12.5%), and seizures (10.9%). HIV was newly diagnosed in 53 patients (82.8%). All 11 patients previously diagnosed with HIV were taking ART, and 6 were using co-trimoxazole as *Pneumocystis jiroveci* (PJP) prophylaxis at time of presentation. The median CD4 cell count was 22 cells/mL, and less than 200 cells/mL in 22 out of 23 patients tested (96%).

CSF *T. gondii* PCR was positive in 21 of 64 HIV-infected patients (32.8%), with a median Ct-value of 36.0 (IQR: 34.2–39.3). None of the 22 HIV-negative control and 9 non-infectious CNS disease patients had a positive *T. gondii* PCR. Archived serum sample was not available in 14 patients. Toxoplasma IgG was positive in 78% of patients tested, including all patients with positive CSF *T. gondii* PCR. Toxoplasma IgG titers were higher among patients with a positive CSF *T. gondii* PCR (p = .017). A definite diagnosis of TB meningitis was established in 21/64 patients (32.8%). Out of 21 patients with positive *T. gondii* PCR, five had combined tuberculosis and toxoplasmosis. Cryptococcosis was diagnosed in 15/64 patients (23.4%), including two who were also diagnosed with tuberculosis. In 14 patients (21.9%) no causative pathogen was isolated.

Neck stiffness, headache and fever, the classical signs of meningitis, were equally common in patients diagnosed with toxoplasmosis, cryptococcosis and tuberculosis, as were most other signs and symptoms, except hemiparesis (**Table 1**). None of the patients with toxoplasmosis had received ART or co-trimoxazole prophylaxis prior to admission with meningitis. CT scans were available for 6 patients, including 4 with a positive *T. gondii* PCR. Three showed signs of hydrocephalus, one a hypodense lesion that showed no enhancement using contrast, and two were normal. No mass lesions typical for cerebral toxoplasmosis were seen.

**Table 1:** Characteristics of patients according to microbiological diagnosis.

	Tuberculosis (n = 14)	Cryptococcosis (n = 13)	Toxoplasmosis (n = 16)
**General**			
Age in year – median (IQR)	30 (27–34)	30 (27–32)	31 (28–33)
Male sex – no. (%)	11 (78.6)	11 (84.6)	11 (68.8)
History of TB treatment – no. (%)	3 (21.4)	3 (23.1)	4 (25.0)
Duration of illness, days – median (IQR)	14 (6–28)	7 (2–14)	7 (4–12)
ART prior to admission – no. (%)	2 (14.3)	4 (30.8)	1 (6.3)
PJP-prophylaxis – no. (%)	0	3 (23.1)	0
**Signs and symptoms on presentation**			
Headache – no. (%)	10/12 (83.3)	10/11 (90.9)	10/12 (83.3)
Seizure – no. (%)	0/13 (0.0)	2/11 (18.2)	2/13 (15.4)
Neck stiffness – no. (%)	12/13 (92.3)	9/11 (81.8)	13/16 (81.3)
Body temperature ≥38°C – no. (%)	4/11 (36.4)	1/8 (12.5)	4/13 (30.8)
Altered consciousness (GCS<14) – no. (%)	6/10 (60.0)	2/10 (20.0)	4/10 (40.0)
GCS – median (range)	13 (6–15)	15 (9–15)	14 (12–15)
Hemi- or tetraparesis – no. (%)	8 (57.1)	0/12 (0.0)	6 (37.5)
Cranial nerve palsy – no. (%)	1 (7.1)	2 (15.4)	3 ( 18.8)
**CSF – median (IQR)**			
Leukocytes – cells/mL*	76 (6–272)	46 (24–188)	14 (2–26)
Percentage of lymphocytes	49 (16–70)	70 (58–96)	70 (50–100)
Protein – g/dL	140 (87–235)	151 (105–245)	130 (90–300)
CSF∶blood glucose ratio **	0.13 (0.06–0.43)	0.20 (0.09–0.41)	0.40 (0.33–0.53)
**Blood examination**			
CD4 cell count (cells/mL) – range ***	2–98	4–45	9–30
Titer of toxo-IgG >1∶300	5/9 (55%)	3/10 (30%)	11/11 (100%)
**Radiological Examination**			
Abnormal Chest X-ray	8/11 (66.7%)	4/9 (44.4%)	5/10 (50.0%)

Patients with combined TB-toxoplasmosis (n = 5), combined TB-Cryptococcus (n = 2), and no definite diagnosis (n = 14) were excluded from the table. Data are presented as no. of patients/no. evaluated (%) unless stated otherwise.

GCS = Glasgow Coma Scale.

* significantly different: TB vs. toxoplasmosis (p = .01); cryptococcosis vs. toxoplasmosis (p = .02).

** significantly different: TB vs. toxoplasmosis (p = .03), cryptococcosis vs. toxoplasmosis (p = .02).

*** CD4 cell counts were only available for 16 patients with definite diagnoses.

**Figure 1:**
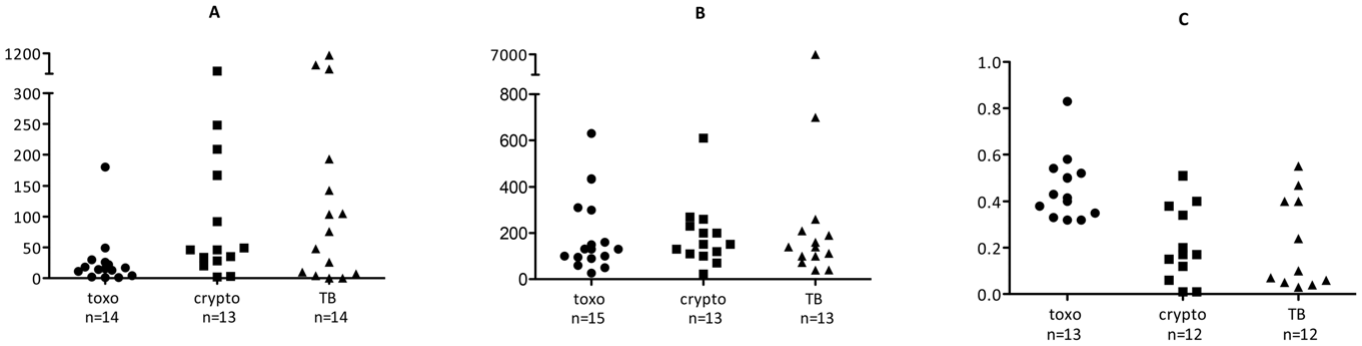
CSF characteristics according to causative pathogen. CSF cell count (A), protein concentration (B) and CSF∶blood glucose ratio (C) for cases with confirmed toxoplasmosis (•), crypotoccococis (▪), and tuberculosis (**▴**). Toxo = toxoplasmosis; crypto = cryptococcosis; TB = TB meningitis.

CSF cell count and protein were normal or mildly elevated in patients with toxoplasmosis, and hypoglycorrhachia was less common compared with tuberculosis or cryptococcosis (**Table 1**). CD4 counts, missing in two-thirds of patients due to early death or the unavailability of CD4 cell testing during the initial phase of the cohort study, were low in all but one patient. **Table 2** lists the CSF findings of individual patients, **Figure 1** is a graphic representation of the CSF cell count, protein and glucose ratio, showing the overlap in CSF findings between patients with toxoplasmosis, cryptococcal and tuberculosis CNS infection.

**Table 2:** Line list of all patients.

No	Age	Sex	CSF examination*			Causative pathogen			Diagnosis**
			Cell number	Protein	Glucose ratio	Toxoφ	TBε	Cryptoν	
1	31	M	4	26	0.33	pos	neg	neg	toxoplasmosis
2	30	M	17	100	0.35	pos	neg	neg	toxoplasmosis
3	33	M	18	160	n/a	pos	neg	neg	toxoplasmosis
4	18	F	11	150	n/a	pos	neg	neg	toxoplasmosis
5	29	F	2	90	0.32	pos	neg	neg	toxoplasmosis
6	24	F	1	300	0.54	pos	neg	neg	toxoplasmosis
7	28	M	13	630	0.4	pos	neg	neg	toxoplasmosis
8	19	F	22	310	0.33	pos	neg	neg	toxoplasmosis
9	30	M	49	130	0.83	pos	neg	neg	toxoplasmosis
10	36	M	1	60	0.58	pos	neg	neg	toxoplasmosis
11	33	M	30	434	0.32	pos	neg	neg	toxoplasmosis
12	35	F	14	95	0.52	pos	neg	neg	toxoplasmosis
13	32	M	26	131	0.5	pos	neg	neg	toxoplasmosis
14	30	M	180	100	0.43	pos	neg	neg	toxoplasmosis
15	34	M	0	50	0.38	pos	neg	neg	toxoplasmosis
16	32	M	n/a	n/a	n/a	pos	neg	neg	toxoplasmosis
17	25	F	143	100	0.13	neg	pos	neg	TB
18	31	M	696	39	0.07	neg	pos	neg	TB
19	30	M	7	260	0.06	neg	pos	neg	TB
20	21	F	105	700	0.05	neg	pos	neg	TB
21	52	M	193	190	0.55	neg	pos	neg	TB
22	29	M	1120	n/a	0.03	neg	pos	neg	TB
23	32	M	104	160	0.04	neg	pos	neg	TB
24	30	M	48	40	0.24	neg	pos	neg	TB
25	28	M	507	6980	0.1	neg	pos	neg	TB
26	23	F	0	73	0.4	neg	pos	neg	TB
27	31	M	26	140	n/a	neg	pos	neg	TB
28	40	M	4	112	0.47	neg	pos	neg	TB
29	56	M	0	100	0.4	neg	pos	neg	TB
30	29	M	10	210	n/a	neg	pos	neg	TB
31	31	M	109	120	0.64	pos	pos	neg	TB-toxo
32	39	M	919	630	0.04	pos	pos	neg	TB-toxo
33	22	M	13	10	0.25	pos	pos	neg	TB-toxo
34	31	M	0	200	0.43	pos	pos	neg	TB-toxo
35	23	F	23	64	0.46	pos	pos	neg	TB-toxo
36	27	M	34	200	0.43	neg	neg	pos	Cryptococcosis
37	30	M	2	22	0.01	neg	neg	pos	Cryptococcosis
38	27	M	419	110	0.06	neg	neg	pos	Cryptococcosis
39	30	M	167	120	0.2	neg	neg	pos	Cryptococcosis
40	38	M	20	230	0.12	neg	neg	pos	Cryptococcosis
41	32	M	209	610	0.01	neg	neg	pos	Cryptococcosis
42	25	F	28	70	0.4	neg	neg	pos	Cryptococcosis
43	32	M	49	100	0.15	neg	neg	pos	Cryptococcosis
44	36	M	46	270	0.51	neg	neg	pos	Cryptococcosis
45	29	M	3	200	0.17	neg	neg	pos	Cryptococcosis
46	22	F	35	151	0.38	neg	neg	pos	Cryptococcosis
47	32	M	248	130	n/a	neg	neg	pos	Cryptococcosis
48	32	M	92	260	0.34	neg	neg	pos	Cryptococcosis
49	27	M	0	n/a	0.16	neg	pos	pos	TB-crypto
50	34	M	0	120	0.27	neg	pos	pos	TB-crypto
51	30	F	0	44	0.39	neg	neg	neg	unknown
52	27	M	0	180	0.49	neg	neg	neg	Unknown
53	33	M	259	110	0.57	neg	neg	neg	Unknown
54	32	M	2	130	0.48	neg	neg	neg	Unknown
55	30	M	n/a	n/a	n/a	neg	neg	neg	Unknown
56	28	M	143	9850	0.04	neg	neg	neg	Unknown
57	30	M	146	40	0.19	neg	neg	neg	Unknown
58	36	M	17	16	0.51	neg	neg	neg	Unknown
59	24	M	0	30	0.6	neg	neg	neg	Unknown
60	29	M	43	44	0.21	neg	neg	neg	Unknown
61	39	M	18	n/a	0.24	neg	neg	neg	Unknown
62	32	M	184	480	0.08	neg	n/a	neg	Unknown
63	28	M	298	317	0.34	neg	neg	neg	Unknown
64	34	M	166	133	n/a	neg	n/a	neg	Unknown

M = male; F = female; pos = positive; neg = negative; n/a = not available.

* cell numbers are in cells/mL; protein in mg/dL; glucose ratio = CSF glucose∶blood glucose.

** TB-toxo = tuberculosis and toxoplasmosis, TB-crypto = tuberculosis and cryptococcosis.

φ toxoplasmosis, based on toxoplasma PCR;

ε TB meningitis, based on either culture or real time PCR;

ν cryptococcosis based on either direct staing, culture, or antigen testing.

**Figure 2:**
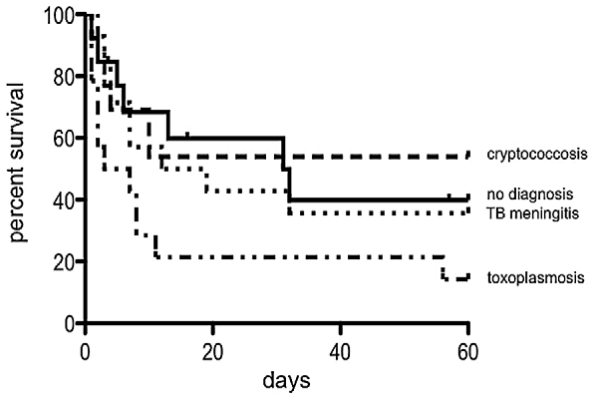
Kaplan Meier survival estimates. Patients with available long term follow up data: Toxoplasmosis (n = 14), Cryptococcosis (n = 13), TB meningitis (n = 14), no diagnosis (n = 13).

Patients with confirmed cryptococcosis received amphotericine B, followed by fluconazole; all others received empiric tuberculosis treatment combined with adjunctive corticosteroids [13]. No toxoplasmosis treatment was given, as *T. gondii* PCR was performed retrospectively and was not available at time of presentation. Eight patients were lost to follow up and were not included in Kaplan Meier analysis. Mortality among those with positive CSF *T. gondii* PCR was 2.16-fold (95% CI 1.04–4.47) higher compared to those who had a negative PCR result; median survival was 7 days for toxoplasmosis, 7 days for tuberculosis meningitis, 110 days for cryptococcosis, and 32 days for patients with an unknown cause of meningitis (**Figure 2**).

## Discussion

In our cohort of HIV-infected patients presenting with clinical signs and symptoms of CNS infection, CSF *T. gondii* PCR was positive in 32.8% of patients, sometimes in conjunction with tuberculosis. In the absence of CT or MRI of the brain, toxoplasmosis could not be distinguished from tuberculosis or cryptococcosis. Mortality in this cohort of newly diagnosed and advanced HIV infection was extremely high and associated with a positive *T. gondii* PCR.

Cerebral toxoplasmosis typically causes space occupying lesion(s), leading to subacute or acutely developing confusion, with or without focal neurological deficits [14]. In the absence of CT or MRI of the brain, common findings like headache, fever, hemiparesis and decreased level of consciousness [10] may mimic those of meningitis [4], [15]–[17]. In previous series of cerebral toxoplasmosis [18], [19], meningeal signs have been reported in 3 to 16% of the patients, although in many reports neck stiffness is not mentioned [14]. Although rare, cases of spinal cord toxoplasmosis have also been reported [20]. No typical mass lesions were found in 6 patients with an available CT scan. This is not surprising, as this study depended on the availability of CSF samples, that would not have been obtained if typical mass lesions had been found.

We used *T. gondii* PCR for diagnosis of cerebral toxoplasmosis. In previous studies CSF *T. gondii* PCR had a sensitivity of 50–60% to confirm cerebral toxoplasmosis in HIV-infected patients [21], [22]. The sensitivity is possibly higher among patients with meningoencephalitis compared to those with space-occupying lesions only, but this has not been examined. Specificity of *T. gondii* PCR is high, between 97 and 100% [22]–[24]. The positivity rate of 32.8% in our study might therefore be an underestimate, especially in the category of patients in whom no other pathogen was isolated despite extensive microbiological testing.

In our cohort, toxoplasmosis could not be distinguished clinically from tuberculosis and cryptococcosis. From our previous series [4], CSF samples were available for the current study for 36/47 HIV-infected patients. Ten out of 17 patients who were diagnosed with ‘probable TB meningitis’ and ‘unknown’ in the previous study were found to have a positive *T. gondii* PCR (and no bacteriological confirmation of tuberculosis) in the current study. Diagnosis of cerebral toxoplasmosis is usually based on clinical findings and CT or MRI of the brain. However, if cerebral imaging is lacking, toxoplasmosis may not be considered. Positive toxoplasma serology, which has a high sensitivity but very poor specificity, is helpful to exclude but not to confirm cerebral toxoplasmosis, although some reports suggest that high toxoplasma IgG titers are only found in patients with symptomatic toxoplasmosis [25]. Indeed, in our study, patients with a positive *T. gondii* PCR had a higher IgG titers compared to those who had a negative PCR. An autopsy study from India provides further support for the notion that cerebral toxoplasmosis is not always considered; among 233 HIV patients, toxoplasmosis accounted for 6.8% of deaths, but in none of these cases toxoplasmosis had been suspected clinically [26].

The incidence of cerebral toxoplasmosis varies between countries [14] and is related to the seroprevalence of toxoplasmosis in the general population [19], [25]. In the United States, toxoplasma seroprevalence varies from 3% to 30%, whereas in France 73%–90% of the population is infected [10]. Reported seroprevalence rates were varied from 13–31% in the general population, and 45–68% in HIV patients in studies from several developing countries [8], [27], [28]. In our study, 78% of patients had detectable toxoplasma IgG, but this does not reflect the seroprevalence in the general population or among unselected HIV-infected patients, as only meningitis patients were examined.

Mortality in this cohort of patients was very high, higher compared to reported rates in other series [8], [9], [29]. One explanation is that patients mostly presented with advanced and untreated HIV infection. In addition, no toxoplasmosis treatment was provided, as PCR was done retrospectively on archived samples. In our previous study, HIV infection was associated with a 2.5-fold increased mortality among patients presenting with meningitis [4]. Data from the current study suggests that this may is at least in part attributable to a high prevalence of (unrecognized and untreated) toxoplasmosis. Future studies should examine the benefit of timely diagnosis and/or empiric treatment of toxoplasmosis for patients in settings like ours. Empiric treatment for subacute meningitis in HIV-infected patients should probably also include tuberculosis, which is difficult to exclude as culture is slow and microscopy and commercial PCR assays have insufficient sensitivity [30].

Our study suffers from several limitations. Most importantly, no CT or MRI of the brain could be performed. In addition, clinical data, CD4 cell counts and other laboratory parameters were missing in a number of patients. Despite these limitations the data strongly suggest that toxoplasmosis should be included in the differential diagnosis of HIV-infected with clinically suspected subacute meningitis, and that molecular testing or empiric treatment for toxoplasmosis should be considered in these patients, especially if no CT or MRI can be performed. Obviously, timely diagnosis and treatment of HIV will help prevent this severe opportunistic infection.

## Supporting Information

Checklist S1 **STROBE checklist for the manuscript: “Cerebral toxoplasmosis mimicking subacute meningitis in HIV-infected patients; a cohort study from Indonesia”.**
(DOC)
