# Supplementary material for: Cerebral Toxoplasmosis Mimicking Subacute Meningitis in HIV-Infected Patients; a Cohort Study from Indonesia
Source: PLoS Negl Trop Dis. 2013 Jan 10;7(1):e1994. doi: 10.1371/journal.pntd.0001994 (PMC3542116; doi:10.1371/journal.pntd.0001994)
Supplement: Checklist S1 — STROBE checklist for the manuscript: “Cerebral toxoplasmosis mimicking subacute meningitis in HIV-infected patients; a cohort study from Indonesia”. (DOC) [file pntd.0001994.s001.doc]

STROBE Statement—checklist of items that should be included in reports of observational studies

|  | Item No | Recommendation |
| --- | --- | --- |
| **Title and abstract** | 1 | (*a*) Indicate the study’s design with a commonly used term in the title or the abstract |
|  |  | **Cohort study was indicated (page 1 line 1-2)** |
|  |  | (*b*) Provide in the abstract an informative and balanced summary of what was done and what was found  **Abstract provides an informative and balanced summary of what was done (page 2 line 9-12) and what was found (page 2 line 13-20)** |
| Introduction | | |
| Background/rationale | 2 | Explain the scientific background and rationale for the investigation being reported  **Page 4 line 3-17** |
| Objectives | 3 | State specific objectives, including any prespecified hypotheses  **Page 4 line 17-19** |
| Methods | | |
| Study design | 4 | Present key elements of study design early in the paper  **Page 4 line 23-24** |
| Setting | 5 | Describe the setting, locations, and relevant dates, including periods of recruitment, exposure, follow-up, and data collection  **Page 5 line 6-9** |
| Participants | 6 | (*a*) *Cohort study*—Give the eligibility criteria, and the sources and methods of selection of participants. Describe methods of follow-up  **Eligibility criteria are described at page 5 line 7**  **Source of participants: page 4 line 23-24** |
| Variables | 7 | Clearly define all outcomes, exposures, predictors, potential confounders, and effect modifiers. Give diagnostic criteria, if applicable  **Outcome variables are defined: Toxoplasma PCR at page 5 line 27-30, and toxoplasma IgG at page 6 line 5-7**  **Diagnostic criteria are described at page 5 line 9-12** |
| Data sources/ measurement | 8* | For each variable of interest, give sources of data and details of methods of assessment (measurement). Describe comparability of assessment methods if there is more than one group  **Source of data: page 4 line 23-24**  **Details of methods of assessment: page 5 line 16-29** |
| Bias | 9 | Describe any efforts to address potential sources of bias  **Page 5 line 29 until page 6 line 5** |
| Study size | 10 | Explain how the study size was arrived at  **No size calculation was made for this study** |
| Quantitative variables | 11 | Explain how quantitative variables were handled in the analyses. If applicable, describe which groupings were chosen and why  **Page 6 line 9-12** |

| Statistical methods | 12 | (*a*) Describe all statistical methods, including those used to control for confounding  **Page 6 line 9-13** |
| --- | --- | --- |
|  |  | (*b*) Describe any methods used to examine subgroups and interactions  **No subgroups were examined** |
|  |  | (*c*) Explain how missing data were addressed  **No imputation was done, those with missing data were left out** |
|  |  | (*d*) *Cohort study*—If applicable, explain how loss to follow-up was addressed  **There was no loss to follow-up** |
|  |  | (*e*) Describe any sensitivity analyses  **Not performed** |

| Results | | |
| --- | --- | --- |
| Participants | 13* | (a) Report numbers of individuals at each stage of study—eg numbers potentially eligible, examined for eligibility, confirmed eligible, included in the study, completing follow-up, and analysed  **Page 6 line 16-17** |
|  |  | (b) Give reasons for non-participation at each stage  **Page 6 line 16-17** |
|  |  | (c) Consider use of a flow diagram |
| Descriptive data | 14* | (a) Give characteristics of study participants (eg demographic, clinical, social) and information on exposures and potential confounders  **Page 6 line 16-23** |
|  |  | (b) Indicate number of participants with missing data for each variable of interest  **Page 6 line 27 (missing data for toxoplasma IgG); page 7 line 16-17 (missing data for CD4 cell count); page 7 line 25-26 (lost to follow up)** |
|  |  | (c) *Cohort study*—Summarise follow-up time (eg, average and total amount)  **Page 6 line 12** |
| Outcome data | 15* | *Cohort study*—Report numbers of outcome events or summary measures over time  **Page 6 line 25 until page 7 line 4 and Figure 2** |
| Main results | 16 | (*a*) Give unadjusted estimates and, if applicable, confounder-adjusted estimates and their precision (eg, 95% confidence interval). Make clear which confounders were adjusted for and why they were included  **Unadjusted risk of death was mentioned in final paragraph of results (page 7 line 26-27). Given the small number of patients, no adjustment was made to possible confounding factors** |
|  |  | (*b*) Report category boundaries when continuous variables were categorized  **95% CI are provided for HR (page 7 line 27); IQR for all other continuous variables are provided on the Table 1 (page 16-17).** |
|  |  | (*c*) If relevant, consider translating estimates of relative risk into absolute risk for a meaningful time period  **Not done given the size of the study** |
| Other analyses | 17 | Report other analyses done—eg analyses of subgroups and interactions, and sensitivity analyses  **Kaplan Meier estimates: Page 7 line 26-29** |

| Discussion | | |
| --- | --- | --- |
| Key results | 18 | Summarise key results with reference to study objectives  **Page 8 line 2-7** |
| Limitations | 19 | Discuss limitations of the study, taking into account sources of potential bias or imprecision. Discuss both direction and magnitude of any potential bias  **Page 10 line 5-12** |

| Interpretation | 20 | Give a cautious overall interpretation of results considering objectives, limitations, multiplicity of analyses, results from similar studies, and other relevant evidence  **Page 8 line 9 until page 10 line 4** |
| --- | --- | --- |
| Generalisability | 21 | Discuss the generalisability (external validity) of the study results  **Page 10 line 7-11** |

| Other information | | |
| --- | --- | --- |
| Funding | 22 | Give the source of funding and the role of the funders for the present study and, if applicable, for the original study on which the present article is based  **In the financial disclosure** |

*Give information separately for cases and controls in case-control studies and, if applicable, for exposed and unexposed groups in cohort and cross-sectional studies.

**Note:** An Explanation and Elaboration article discusses each checklist item and gives methodological background and published examples of transparent reporting. The STROBE checklist is best used in conjunction with this article (freely available on the Web sites of PLoS Medicine at , Annals of Internal Medicine at http://www.annals.org/, and Epidemiology at http://www.epidem.com/). Information on the STROBE Initiative is available at www.strobe-statement.org.
